# Resveratrol Increases Nephrin and Podocin Expression and Alleviates Renal Damage in Rats Fed a High-Fat Diet

**DOI:** 10.3390/nu6072619

**Published:** 2014-07-14

**Authors:** Qing-Rong Pan, Yan-Long Ren, Jia-Jia Zhu, Yan-Jin Hu, Jin-Su Zheng, Hui Fan, Yuan Xu, Guang Wang, Wen-Xian Liu

**Affiliations:** 1 Department of Endocrinology, Beijing Chaoyang Hospital, Capital Medical University, Beijing 100020, China; E-Mails: panqingrongcn@163.com (Q.-R.P.); longwesthill@163.com (Y.-J.H.); newmoon163@163.com (H.F.); xuyuan32001@sina.com (Y.X.); drwg6688@163.com (G.W.); 2 Department of Cardiology, Beijing Anzhen Hospital, Capital Medical University, Beijing 100029, China; E-Mails: renyanlong@yeah.net (Y.-L.R.); zhujiajia83@126.com (J.-J.Z.); 3 Department of Traditional Chinese Medicine, Beijing Chaoyang Hospital, Capital Medical University, Beijing, 100020, China; E-Mail: rosamand@163.com

**Keywords:** resveratrol, NF-κB, TNF-α, MCP-1, nephrin, podocin

## Abstract

Resveratrol is well known for its anti-inflammation and anti-oxidant properties, and has been shown to be effective in alleviating the development of obesity. The purpose of this investigation was to analyze the effect of resveratrol on renal damage in obese rats induced by a high-fat diet (HFD) and its possible mechanisms. Male Sprague-Dawley rats were divided into three groups: control, HFD, and HFD plus resveratrol (treated with 100 mg/kg/day resveratrol). Body weight, serum and urine metabolic parameters, and kidney histology were measured. Meanwhile, the activities of nuclear factor-κB (NF-κB) and superoxide dismutase (SOD), the content of malondialdehyde (MDA), and the protein levels of tumor necrosis factor (TNF-α), monocyte chemotactic protein-1 (MCP-1), nephrin and podocin in kidney were detected. Our work showed that resveratrol alleviated dyslipidemia and renal damage induced by HFD, decreased MDA level and increased SOD activity. Furthermore, the elevated NF-κB activity, increased TNF-α and MCP-1 levels, and reduced expressions of nephrin and podocin induced by HFD were significantly reversed by resveratrol. These results suggest resveratrol could ameliorate renal injury in rats fed a HFD, and the mechanisms are associated with suppressing oxidative stress and NF-κB signaling pathway that in turn up-regulate nephrin and podocin protein expression.

## 1. Introduction

Studies in the general population suggest that obesity is an independent risk factor for development and progression of chronic kidney disease [1,2]. Obesity itself could induce renal injury, namely, obesity-related glomerulopathy (ORG), which may lead to end-stage renal disease [3,4]. It has also been reported that proteinuria, glomerular hypertrophy and segmental glomerulosclerosis can be present in obese patients, even in the absence of diabetes [3].

Proteinuria is an important manifestation of glomerular injury as well as an independent mediator of progressive kidney damage in obesity [5,6]. Podocytes are highly differentiated epithelial cells constituting critical components of the glomerular filtration barrier through the slit diaphragms [7]. The damage to podocytes results in proteinuria and eventually renal failure [7,8]. Decreased podocyte density and number, correlated with degree of proteinuria and renal function impairment, were observed in patients with ORG [9]. Podocyte proteins, such as nephrin and podocin, are critical for maintaining the structural integrity of slit diaphragm [10,11,12]. Several studies have demonstrated the presence of reduced nephrin and podocin expressions in some proteinuric glomerular diseases [13,14,15].

Resveratrol (*trans*-3,5,4′-trihydroxystilbene) is one of the natural polyphenolic compounds mainly found in grape skin, peanut roots and other various plants. Resveratrol is well known for its anti-inflammatory, anti-oxidant, anti-cancer, and anti-ageing properties [16]. Chronic low-grade inflammation and elevated oxidative stress, which are important features of obesity, have been considered to play a vital role in the pathogenesis and progression of ORG [17]. Recently, resveratrol has been suggested to be effective in preventing the development of obesity and diabetes [18,19]. Moreover, emerging evidence demonstrated that resveratrol could alleviate renal injury in diabetic mice and rats [20,21]. However, the effects of resveratrol on renal damage induced by obesity in absence of diabetes have not been previously explored.

In the present study we investigated the renal function, oxidative stress, pro-inflammatory molecules, and the expression of nephrin and podocin in kidney of rats fed a high-fat diet, a model of obesity. We also explored whether resveratrol had renoprotective effect in rats fed a high-fat diet, and whether this therapeutic approach resulted in a down-regulation of oxidative stress and pro-inflammatory molecules, and changes of nephrin and podocin expression in kidney.

## 2. Materials and Methods

### 2.1. Animals

Six-week-old male Sprague-Dawley (SD) rats weighing approximately 190 g were purchased from the SPF animal center (Beijing, China). The rats were kept in a room with controlled temperature (20–23 °C) and lighting (alternating 12 h periods of light and dark). The animals had *ad libitum* access to water and were fed throughout the trial period. All procedures were approved by the Ethics Committee for Animal Studies at Capital Medical University, China.

### 2.2. Research Design, Diet and Resveratrol Treatment

Initially the animals were fed either a control diet (*n* = 10) or a HFD (*n* = 20) for 4 weeks. The control diet was a normal chow diet (Beijing HFK Bioscience, Beijing, China) composed of a balanced level of protein, carbohydrate, and fat in the proportion 20%/70%/10% of total calories, respectively. The composition of the HFD (Research Diet, New Brunswick, NJ, USA) was changed to 20%/35%/45% of total calories. The normal chow diet contained 3.85 kcal/g, and the HFD contained 4.73 kcal/g. The HFD-induced obese model was based on the model reported by Dube *et al*. [22]. After 4 weeks, the rats fed the HFD were randomly distributed into two groups of 10 rats each: HFD group and HFD plus resveratrol (HFD + Res) group. The rats fed the control diet were classified as the control(C) group. The Rats in the HFD + Res group were orally administered resveratrol (100 mg/kg/day, Sigma, St. Louis, MI, USA) using gastric gavage. The Rats of C and HFD groups were given the same volume of normal saline. Body weights of the rats of all three groups were measured weekly. The rats were fed and treated as such for the next 8 weeks. Oral glucose tolerance test (OGTT) was performed at the 11th week on the feeding regime. At the end of the experiment, after an overnight fasting, the animals were euthanized by using an intraperitoneal injection of chloral hydrate. Blood was collected, and epididymal and omental fat depots were removed and weighed. Kidneys were dissected, weighed, and immediately frozen or fixed in a buffer solution of 10% formalin.

### 2.3. Oral Glucose Tolerance Test (OGTT)

Rats received an oral glucose dose of 3 g/kg by gavage after fasting for 12 h. Then blood samples were taken before the oral glucose dose (0 min) and at the following time points: 30 min, 60 min, and 120 min. Blood samples were drawn by tail prick and immediately measured by blood glucose meter with the glucose oxidase peroxidase method (Bayer, Germany). AUC was calculated for blood glucose during the OGTT:

AUC = 0.5 × [BG0 + BG30]/2 + 0.5 × [BG30 + BG60]/2 + [BG60 + BG120]/2
(1)

### 2.4. Biochemical Analysis

Commercially available enzyme-linked immunosorbent assay kits were used for the analysis: serum insulin (Millipore-linco, Boston, MA, USA), serum total cholesterol (TC) (Cusabio, Wuhan, China) and triacylglycerol (TG) (Cusabio, Wuhan, China). HOMA-IR index was calculated according to the following formula:

HOMA-IR = fasting blood glucose (mmol/L) × fasting serum insulin (μU/mL)/22.5
(2)

### 2.5. Assessment of Renal Function

The 24-h urine samples were collected at the 12th week. The rats were housed in metabolic cages (Harvard Apparatus, Holliston, MA, USA) for 24 h to collect urine samples. Urine volumes were measured and samples were centrifuged at 3000× *g* for 10 min to remove any suspended particles and were stored in aliquots at −80 °C. Creatinine levels and urine protein were measured with an Olympus AU 5400 analyzer (Olympus Diagnostica, Hamburg, Germany). Urinary protein was determined by the sulfosalicylic acid method. Creatinine level was measured by creatinine enzymatic assay. Creatinine clearance rate (Ccr) was calculated using the following formula [23]:

Ccr (mL/min/kg body weight) = [urinary creatinine (mg/dL) × urine volume (mL) × 1000]/[serum creatinine (mg/dL) × body weight (g) × 1440 (min)]
(3)

and was expressed as milliliter per minute per kilogram.

### 2.6. Renal Histological Analysis

All fixed kidney tissues were processed routinely for paraffin embedding and 4 mm sections were prepared, stained with periodic acid-Schiff (PAS) and examined under light microscopy. The glomerular cross-sectional area (Ag) was measured in 30 glomerular profiles per rat by using NIS-Elements 3.2 (Nikon Corporation, Tokyo, Japan). Glomerular extracellular matrix (ECM) area was defined as the PAS-positive area. The ratio of glomerular ECM area to Ag was also calculated to obtain the relative area of the glomerular ECM. The glomerular volume (Vg) was determined as: Vg = β/κ [Ag]^3/2^, where β is 1.38 as a shape factor and κ is 1.1 as a distribution factor [24].

### 2.7. NF-κB DNA-Binding Activity, SOD Activity, and MDA Level in Kidney

Nuclear proteins were extracted from the kidney cortical tissues with a Nuclear Extract Kit (Cayman chemical, Ann Arbor, MI, USA). NF-κB DNA-binding activity was measured by NF-κB (p65) Transcription Factor Assay Kit (Cayman chemical, Ann Arbor, MI, USA) according to the manufacturer’s instructions. MDA level and SOD activity were quantified in kidney cortical tissues using Cayman’ assay kit (Cayman Chemical, Ann Arbor, MI, USA).

### 2.8. Western Blot

The kidney cortical tissues were homogenized in RIPA lysis buffer (1% Triton X-100, 1% deoxycholate, 0.1% SDS). Protein concentration was determined by Bradford method. Aliquots of 40–60 μg of protein were separated by SDS-PAGE (10% resolving gel), transferred to PVDF membranes (Millipore, Boston, MA, USA), and blocked with 10% nonfat milk. Membranes were incubated with primary antibodies overnight at 4 °C. The following primary antibodies were applied: anti-nephrin (1:400, Abcan, Cambridge, UK), anti-podocin (1:400, Abcan, Cambridge, UK), anti-TNF-α (1:300, Santa Cruz, CA, USA), and anti-MCP-1 (1:300, Santa Cruz, CA, USA). Primary antibodies were detected by secondary antibodies (1:2000, Santa Cruz, CA, USA). The protein bands were visualized with chemiluminescence reagent (ECL, Cell Signaling, Beverly, MA, USA) and quantified using Scion Image software. After determination of target proteins, membranes were incubated in stripping buffer (0.2 mmol/L glycine, 0.02% Tween 20, PH 7.5; 20 min at 80 °C), washed extensively, and subjected to immunoblotting analysis to determine β-actin (1:2500, Santa Cruz, CA, USA).

### 2.9. Statistical Analyses

All data were presented as means ± standard error (SD), and analyses were performed using SPSS 16.0 statistical software. Differences between groups were analyzed by one way ANOVA. The LSD *post hoc* test was used if ANOVA was significant. When necessary, we used the nonparametric Kruskal-Wallis test or Mann-Whitney rank test, respectively. The statistical value of *p* < 0.05 was considered as statistically significant.

## 3. Results

### 3.1. Effects of Resveratrol on Body Weight, Kidney Weight, and Omental and Epididymal Fat Weights

Body weight gains of rats were increased significantly by HFD feeding. After eight weeks of resveratrol treatment, the weights of rats in the HFD + Res group appeared lower than those in the HFD group, and the difference, although not statistically significant (628.6 ± 17.28 g *vs.* 652.8 ± 29.66 g, *p* = 0.055), was close to statistical significance (Table 1). Kidney weight was increased by HFD-feeding, but resveratrol treatment did not affect kidney weight significantly (Table 1). On the other hand, resveratrol significantly decreased the epididymal and omental fat weights which were elevated by HFD (Table 1).

**Table 1:** Effects of resveratrol on body weight, food and energy intake and organ weights.

Parameters	C	HFD	HFD + Res	*p* value	
				HFD *vs.* C	HFD + Res *vs.* HFD
Initial body weight (g)	189.98 ± 6.58	187.89 ± 7.69	189.2 ± 7.71	0.53	0.69
Final body weight (g)	519.16 ± 31.75 ^a^	652.8 ± 29.66 ^b^	628.6 ± 17.28 ^b^	<0.001	0.055
Food intake at 12 weeks (g/day)	27.2 ± 3.0	29.9 ± 3.3	28.6 ± 3.8	0.09	0.40
Energy intake at 12 weeks (kcal/day)	104.72 ± 11.55 ^a^	141.43 ± 15.61 ^b^	135.28 ± 14.97 ^b^	<0.001	0.81
Kidney weight (g)	2.95 ± 0.30 ^a^	3.52 ± 0.41 ^b^	3.22 ± 0.28 ^b^	0.001	0.053
Relative kidney weight (mg/mm tibial length)	55.02 ± 2.44 ^a^	64.41 ± 4.46 ^b^	60.54 ± 4.45 ^b^	<0.001	0.61
Epididymal fat weight (g)	6.20 ± 0.48 ^a^	15.53 ± 1.01 ^b^	13.74 ± 1.64 ^c^	<0.001	0.002
Relative epididymal fat weight (mg/mm tibial length)	117.14 ± 11.08 ^a^	267.91 ± 17.05 ^b^	232.93 ± 24.62 ^c^	<0.001	0.001
Omental fat weight (g)	3.74 ± 0.26 ^a^	8.06 ± 0.22 ^b^	7.72 ± 0.20 ^c^	<0.001	0.02
Relative omental fat weight (mg/mm tibial length)	65.59 ± 3.07 ^a^	139.94 ± 2.4 ^b^	136.21 ± 3.3 ^c^	<0.001	0.04

Data are presented as the mean ± SD; *n* = 10; values with different superscript alphabets in the same row are significantly different (*p* < 0.05).

### 3.2. Effects of Resveratrol on Blood Glucose Level, Serum Lipid Level, and Insulin Resistance

**Figure 1:**
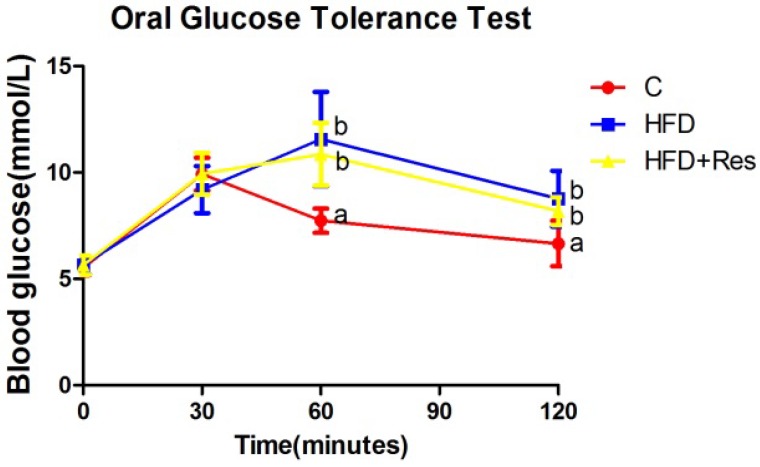
Effect of resveratrol on oral glucose tolerance. Blood glucose levels at 60 and 120 min within OGTT in the HFD and HFD + Res groups were higher than the C group, but no significant difference was observed between the former two groups. Data are presented as the mean ± SD; *n* = 10; values with different superscript alphabets in the same row are significantly different (*p* < 0.05).

Blood glucose levels in three groups were elevated after the administration of glucose to rats. Blood glucose levels at 0 and 30 min within OGTT were not significantly different among three groups (Figure 1). Blood glucose levels at 60 and 120 min within OGTT, AUC, serum fasting insulin level and HOMA-IR in the HFD and HFD + Res groups were higher than the C group, but no significant difference was observed between the former two groups (Figure 1, Table 2). The increased serum TG and TC concentrations in rats fed with HFD were decreased by resveratrol (Table 2).

**Table 2:** Effects of resveratrol on blood glucose, serum insulin, serum lipid, creatinine clearance rate, and urinary protein excretion.

Parameters	C	HFD	HFD + Res	*p* value	
				HFD *vs.* C	HFD + Res *vs.* HFD
Fasting blood glucose (mmol/L)	6.39 ± 0.42	6.76 ± 0.42	6.69 ± 0.48	0.07	0.74
Fasting serum insulin (ng/mL)	0.84 ± 0.07 ^a^	1.29 ± 0.11 ^b^	1.24 ± 0.27 ^b^	<0.001	0.53
HOMA-IR	5.15 ± 0.99 ^a^	8.20 ± 0.33 ^b^	8.03 ± 0.32 ^b^	<0.001	0.57
OGTT(AUC)	663.7 ± 15.97 ^a^	688.2 ± 16.14 ^b^	692.5 ± 19.31 ^b^	0.004	0.58
TG (mmol/L)	0.82 ± 0.13 ^a^	5.15 ± 1.23 ^b^	2.32 ± 0.40 ^c^	<0.001	<0.001
TC (mmol/L)	1.91 ± 0.17 ^a^	3.08 ± 0.43 ^b^	1.93 ± 0.24 ^a^	<0.001	<0.001
Serum creatinine (μmol/L)	29.69 ± 3.99 ^a^	36.93 ± 5.69 ^b^	34.53 ± 5.03 ^b^	0.003	0.29
Ccr (mL/min/kg body weight)	52.19 ± 3.72 ^a^	44.62 ± 2.65 ^b^	47.67 ± 2.07 ^c^	<0.001	0.03
Urinary protein excretionμg (per 24 h)	457.55 ± 127.77 ^a^	1430.88 ± 359.80 ^b^	909.32 ± 80.07 ^c^	<0.001	0.001

TG: triglyceride; TC: tolal-cholesterol; HOMA-IR: homeostatic model assessment-insulin resistance; OGTT (AUC): oral glucose tolerance (area under curve); Ccr: creatinine clearance rate. Data are presented as the mean ± SD; *n* = 10; values with different superscript alphabets in the same row are significantly different (*p* < 0.05).

### 3.3. Protective Effects of Resveratrol on Proteinuria and Renal Damage Induced by HFD

**Figure 2:**
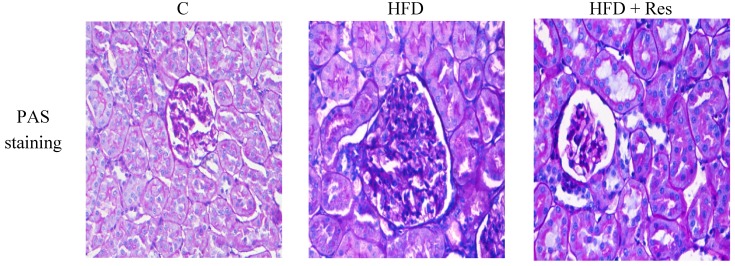
Histopathological observation of kidney tissue in the rats (PAS staining, original magnification: 400×). The increase in glomerular volume and more accumulation of glomerular ECM were observed in HFD-fed rats. Treatment of resveratrol alleviated these histological changes.

Serum creatinine level was increased by HFD-feeding (*p* < 0.05), but resveratrol treatment did not affect serum creatinine level significantly. A significant decrease of creatinine clearance, and an obvious elevation in urine protein (2.74-fold) were induced by HFD. Resveratrol treatment partially reversed these HFD-induced disturbances (Table 2). The control group had normal histology, while enlarged glomerular volume and increased vacuolar degeneration in renal tubules were revealed in the HFD group (Figure 2 and Table 3). Moreover, there was also significantly more accumulation of glomerular ECM in the HFD group. Treatment with resveratrol clearly alleviated these histological changes induced by HFD (Figure 2 and Table 3).

**Table 3:** Glomerular volume and glomerular ECM accumulation.

Parameters	C	HFD	HFD + Res	*p* value	
				HFD *vs.* C	HFD + Res *vs.* HFD
Glomerular volume (10^6^ μm^3^)	0.92 ± 0.19 ^a^	1.47 ± 0.31 ^b^	1.13 ± 0.27 ^c^	0.01	0.02
ECM Accumulation (arbitrary units)	0.074 ± 0.003 ^a^	0.113 ± 0.004 ^b^	0.098 ± 0.002 ^c^	0.01	0.01

ECM: extracellular matrix; ECM Accumulation (arbitrary units) = The area of the glomerular ECM/glomerular cross-sectional area. Data are presented as the mean ± SD; *n* = 10; values with different superscript alphabets in the same row are significantly different (*p* < 0.05).

### 3.4. Resveratrol Restored the Protein levels of Nephrin and Podocin in Kidney

**Figure 3:**
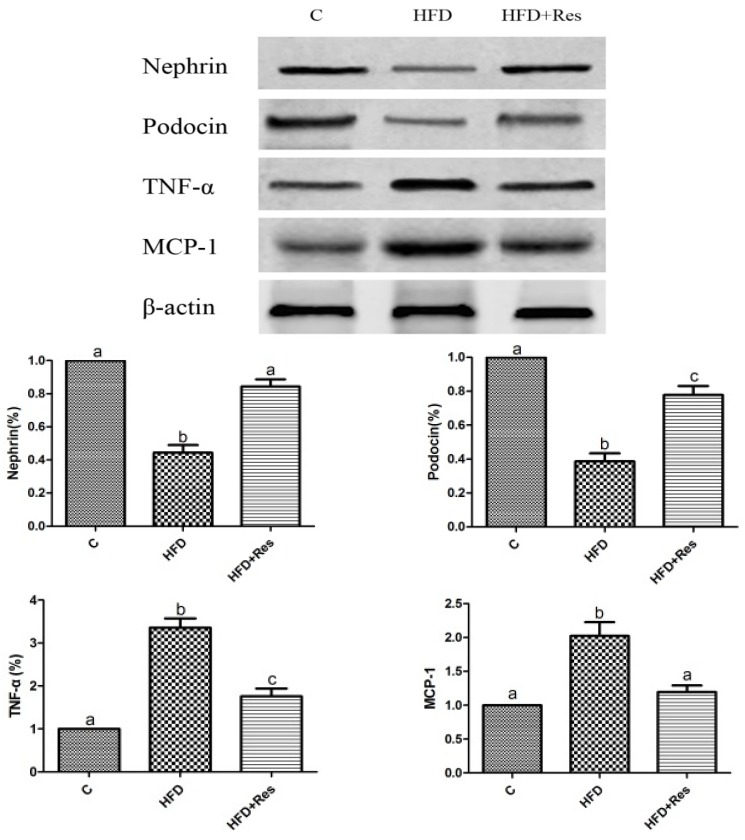
Effects of resveratrol on the protein level in kidney. Data are presented as mean ± SD; *n* = 10; values with different superscript alphabets in the same row are significantly different (*p* < 0.05).

Western blot assay indicated that the renal nephrin and podocin proteins were less expressed in the HFD group compared to that in the control group. Furthermore, 8 weeks resveratrol treatment alleviated HFD-induced loss of glomerular nephrin and podocin expressions (Figure 3).

### 3.5. Resveratrol Reduced Renal NF-κB Activity, TNF-α and MCP-1 Levels, and Alleviated Renal Oxidative Stress Induced by HFD

The elevated renal NF-κB activity (Table 4) and increased TNF-α and MCP-1 levels in HFD-fed rats were reduced by resveratrol (Figure 3). To evaluate renal oxidative stress, we measured the MDA level and SOD activity in kidney. Renal MDA was significantly increased by HFD feeding. Resveratrol treatment decreased MDA level (Table 4). The decreased SOD activity in rats fed with HFD was significantly increased by resveratrol (Table 4).

**Table 4:** Renal oxidative stress and NF-κB activity.

Parameters	C	HFD	HFD + Res	*p* value	
				HFD *vs.* C	HFD + Res *vs.* HFD
Renal MDA (nmol/mg)	4.71 ± 0.81 ^a^	12.20 ± 1.44 ^b^	7.89 ± 0.83 ^c^	<0.001	<0.001
Renal SOD activity (U/mg)	23.88 ± 2.74 ^a^	7.46 ± 1.54 ^b^	18.77 ± 3.23 ^c^	<0.001	<0.001
Renal NF-κB activation assay (OD at 450 nm)	0.35 ± 0.04 ^a^	1.07 ± 0.02 ^b^	0.64 ± 0.05 ^c^	<0.001	<0.001

MDA: malondialdehyde; SOD: superoxide dismutase; NF-κB: nuclear factor-κB. Data are presented as the mean ± SD; *n* = 10; values with different superscript alphabets in the same row are significantly different (*p* < 0.05).

## 4. Discussion

At the clinical level, ORG typically manifests with overt proteinuria in obese patients without any other defined primary or secondary glomerular diseases [3,4]. Histologically, ORG presents as focal segmental glomerulosclerosis (FSGS) and glomerular hypertrophy or glomerular hypertrophy alone [3]. In the present study, we showed that HFD provoked an overt obesity characterized by increased body weight gain, fat accumulation, hyperinsulinemia, and dyslipidemia. We also found that HFD induced renal damage as evidenced by elevated serum creatinine level, reduction of creatinine clearance, obvious proteinuria, glomerular hypertrophy, vacuolar degeneration in renal tubules, and increased glomerular ECM accumulation. These data indicated that the ORG model presenting proteinuria, renal glomerular hypertrophy and renal dysfunction was successfully created.

We found that 8 weeks resveratrol treatment improved creatinine clearance and decreased proteinuria. In addition, resveratrol attenuated glomerular hypertrophy, glomerular ECM accumulation and vacuolar degeneration in renal tubules induced by HFD. All these results indicated resveratrol treatment attenuated renal damage induced by HFD. Previously, Charradi *et al.* [25] observed grape seed and skin extract alleviated high-fat diet-induced renal lipotoxicity in rats. But the authors did not identify the exact active ingredient in the grape seed and skin extract that produced this effect. To the best of our knowledge, our work is the first to demonstrate the protective effect of resveratrol on nephrotoxicity in HFD induced obese animal model without diabetes, suggesting a novel treatment of resveratrol in ORG patients.

Recently, the identification of some novel molecules constituting the slit diaphragm along with the description of their function has brought in a new insight into the mechanism of proteinuria in chronic kidney diseases. Nephrin is the first identified structural protein of the podocyte slit diaphragm [26]. Nephrin forms the scaffold for intertwining other slit molecules and plays a pivotal role in preventing passage of protein through the glomerular barrier [11]. Podocin is a stomatin-like protein exclusively expressed in podocytes and an important intracellular binding partner of nephrin [12]. Mutations in the podocin gene cause massive proteinuria and severe podocyte structural alterations [27,28]. Moreover, the expressions of nephrin and podocin have been shown to be reduced in various proteinuric renal diseases [13,14,15]. The present study showed that nephrin and podoxin expressions were reduced in rats with HFD-induced obesity. We also observed that administration of resveratrol led to a significant reversal of proteinuria, accompanied by restoration of nephrin and podocin protein expressions. The restored expressions of these podocytes proteins probably contributed to attenuation of glomerular barrier dysfunction and massive proteinuria induced by HFD.

The pathophysiology of ORG remains incompletely understood. Potential mechanisms by which obesity affects renal physiology include altered renal hemodynamics, insulin resistance, hyperlipidemia, inflammation, oxidative stress and activation of the renin angiotensin-aldosterone system [29]. Our data showed that resveratrol attenuated hyperlipidemia and fat accumulation induced by the HFD, although this treatment did not reduce body weight and insulin resistance significantly. The attenuation of hyperlipidemia and fat accumulation may contribute to alleviative the effects of resveratrol on renal damage.

It is well known that inflammation and oxidative stress play important roles in podocyte injury and the downregulation of nephrin and podocin [30]. MDA is a marker of lipid peroxidation induced by oxidative stress. And SOD is an important defensive metalloproteinase which catalyzes the dismutation of superoxide radicals [17]. In the present study, the increased MDA level and decreased SOD activity in kidney, indicating increased oxidative stress, were noticed in HFD-induced obese rats. Furthermore, resveratrol treatment reversed the change of MDA level and SOD activity. Oxidative stress could lead to pro-inflammatory cytokine production, which may further increase oxidative stress, setting a vicious cycle [17]. Our study also showed the rise of renal NF-κB activity, TNF-α and MCP-1 level induced by HFD was reversed by resveratrol. Increasing data suggests a pivotal role for NF-κB in the development and progression of chronic kidney diseases. Once activated, NF-κB heterodimers p65/p50 bind to specific DNA sequences to initiate transcription and protein expression, such as TNF-α and MCP-1, leading to significant inflammatory responses [31]. TNF-α, a pro-inflammatory cytokine, is involved in the genesis of inflammation and contributes to renal damage [32]. Gene expression profiles in glomeruli obtained from renal biopsy samples of patients with ORG showed an increase of TNF-α and its receptors, suggesting a role for TNF-α in the development of ORG [33]. TNF-α also has been shown to induce the expression of MCP-1, which is a key regulator in recruiting monocytes to the glomeruli and also contributes to renal damage [34,35]. The suppression of the NF-κB signaling pathway and oxidative stress is likely one of the mechanisms of the renoproctective effect of resveratrol.

## 5. Conclusions

In conclusion, the results obtained in the present study suggest that resveratrol ameliorates renal injury and proteinuria in obese rats induced by a HFD. These effects are at least partly produced by inhibiting the NF-κB signaling pathway and oxidative stress and thereby modulating nephrin and podocin protein expressions. Therefore, these studies suggest that resveratrol may be a novel therapeutic agent for ORG patients.
